# Supplementary material for: Mitochondrial DNA Variation of the Striped Hyena (Hyaena hyaena) in Algeria and Further Insights into the Species’ Evolutionary History
Source: Genes (Basel). 2026 Jan 20;17(1):111. doi: 10.3390/genes17010111 (PMC12840928; doi:10.3390/genes17010111)
Supplement: Supplementary file 1 [file genes-17-00111-s001.zip › genes-4017269-supplementary.pdf]

Supplementary Materials

Methodological details of the demographic history analyses

We constructed skyline plots [1], which do not depend on a prespecified parametric model of demographic history, using the following four MCMC sampling-based methods: Bayesian Skyline Plot (BSP; [2]), Extended Bayesian Skyline Plot (EBSP; [3]), Bayesian Skyride [4], and Bayesian Skygrid [5]. These skyline plot methods were performed in BEAST 1.10.4 ('Bayesian Evolutionary Analysis Sampling Trees'; [6]), using the BEAGLE 3.1.0 library [7], with the data partitioned by codon position. All BEAST input files were created in BEAUti, available in the BEAST package. In the BSP analysis we used four groups and performed analyses with both the piece-wise-constant and piecewise-linear models, and for the substitution rate parameter we tested normal, exponential, and uniform prior distributions. The lengths of the MCMC chains were 200 million generations, sampled every 20000th after a pre-burn-in of 20 million. In the EBSP analysis we performed runs with both the stepwise and linear models, and for the substitution rate parameter we tested normal and uniform prior distributions. A uniform distribution was used for the population prior mean. The lengths of the MCMC chains were 200 million generations, sampled every 20000th after a pre-burn-in of 20 million. For the Skyride model, we used time-aware smoothing, a normal prior distribution for the substitution rate parameter, and MCMC chains with 200 million generations, sampled every 20000th after a pre-burn-in of 20 million. For Skygrid, we used 75 grid points, a cutoff value of 1 Ma, a normal prior distribution for the substitution rate parameter, and MCMC chains with 200 million generations, sampled every 20000th after a pre-burn-in of 20 million. For each of the four types of MCMC sampling-based skyline plot analyses, we used Tracer 1.7.2 [8], with a default burn-in of 10% of the chain length, to assess convergence of the chain to the stationary distribution, plot marginal posterior densities, and obtain estimates and ESS of parameters. To draw the skyline plots we also used Tracer, and the Python script popGraphFromCSV.py (obtained from https://github.com/benb/beast-mcmc/tree/master/doc/EBSP/scripts). We compared different skyline models, and also simple parametric models (such as constant population size or exponential growth) (see [9]), in terms of their relative fit to the data,

using the Bayes factor [10,11], the ratio of the marginal likelihoods of the two models under comparison. The marginal likelihoods were estimated in BEAST with path sampling [12] and stepping-stone sampling [13], based on 100 path steps and a chain length of two million iterations.

We also tested skyline plot methods based on a single tree [1]: classical [14], generalized [15], and Bayesian multiple-change-point (MCP; [16]). The required ultrametric binary tree was a maximum clade credibility tree (MCCT) from an analysis in MrBayes. MCMC in MrBayes was performed as described in the main text. The analysis used a strict clock model with a uniform prior probability distribution on branch lengths, a normally distributed clock rate prior with mean 0.01 and standard deviation 0.0025 (in substitutions per site per Ma) (how we obtained this substitution rate estimate is explained in the main text), and a truncated normal prior probability distribution on the tree age with a mean of 0.4 Ma and a standard deviation of 0.15 (the choice of these values reflects the estimates obtained for the age of the mtDNA diversity, presented in the main text). The single-tree skyline plot analyses were performed in the R package APE version 5.7.1 [17]. The analyses performed in the R environment used R version 4.2.3 [18]. Bayesian MCP analyses were performed assuming both a constant population size prior demographic function [19] and a skyline plot prior demographic function [16]. We also estimated the fit of different demographic models (constant population size, exponential growth, expansion growth, logistic growth, with both continuous and piecewise variants) [20] to the MCCT in the R package genieR version 0.1.0 [21].

We also used the Monte Carlo likelihood approach of Weiss and von Haeseler [22], as implemented in IPHULA 1.16 [23], for inference on demographic history. In this analysis we used the HKY model, which was selected as best-fitting the 648 bp alignment by BIC in ModelFinder and also in jModelTest 2.1.10 [24], and a transition/transversion rate ratio (kappa, κ) of 28 estimated in TREE-PUZZLE 5.3.rc16 [25]. The method of Weiss and von Haeseler [22] involves a model with three parameters (theta, θ: mutation-scaled effective population size (N_e) in the past ('initial'), i.e. before an eventual N_e change; tau, τ: mutation-scaled time since N_e started to change exponentially; rho, ϱ: ratio of current to initial N_e), which are estimated based on the

number of segregating sites and the mean number of pairwise nucleotide differences (k). The method approximates the likelihood surface for the three parameters by coalescent simulations on a three-dimensional grid. After preliminary simulations to explore the likelihood surface and find the parameter ranges that enclose the global maximum likelihood peak, we ran 10 replicate runs, zoomed into grid areas of high likelihoods, for parameter estimation. In these final runs we defined five grid points, equally spaced between 1 and 20, for ϱ, 10 grid points, equally spaced between 0.1 and 5, for θ, and 10 grid points, equally spaced between 0.1 and 2, for τ. We ran 50,000 simulations for each grid point (i.e. parameter combination), and the maximum allowed difference between simulated and observed k was set to less than 0.03 (i.e. ± 1% around the observed mean k of 1.72).

Inference on demographic history parameters was also performed with the MCMC coalescent genealogy sampler LAMARC 2.1.10 [26,27], assuming either an exponential growth or shrinkage model or a simpler model of constant population size. We used the Bayesian version of LAMARC, with a default starting value of θ per site of 0.01 and a logarithmic prior between 10^{-5} and 10; in the growth model analysis, we specified for the growth rate parameter (g) a starting value of 1 and a linear prior bounded between -500 and 5000. Percentile profile likelihoods were computed for both θ per site and g. The HKY model is not implemented in LAMARC, so we used the closest model available, Felsenstein84 (F84; [28]), which in any case is very closely related to the HKY model. We ran four replicates of two final chains, in which for each chain we used an initial burn-in period of 200,000 discarded genealogies, followed by two million genealogies sampled every 50 for parameter estimation. We used adaptive heating, with four tree search temperatures (initial values: 1, 1.2, 1.5, 2) in each chain, and tree swapping between temperatures attempted at every step of the chain. Tracer 1.7.2 [8] was used to analyse the resulting trace files, assess run convergence, and calculate summary statistics and ESS values of parameters.

We also tested constant population size and exponential growth models in Genetree 9.01, which implements a Markov chain technique for ancestral inference that simulates gene trees conditional on the mutation pattern in a sample of DNA sequences [29,30,31,32]. Genetree requires data compatible with the infinite sites model [32,33].

To meet this requirement, in the 648 bp dataset we needed to remove one polymorphic site (site 546) inconsistent with the model, which caused the haplotypes H1 and H3, as well as H7 and H10, to no longer differ from each other. In the constant population model, after preliminary runs with different values of the generating parameter for θ to optimize settings and identify the region of maximum likelihood of θ, we generated likelihood surfaces for θ based on 10 million simulations and 1000 points for the empirical distribution. In the exponential growth model, after preliminary runs to identify the likelihood surface regions of θ and growth rate yielding the highest joint likelihood, as the two parameters are not independent of each other, we generated likelihood surfaces separately for θ and growth rate, in both cases based on 100,000 simulations and 1000 points for the empirical distribution. A likelihood ratio test (LRT) was used to determine whether there was a significant difference in the fit to the data between the simpler constant population size model and the exponential growth model.

References

1. Ho, S. Y. W.; Shapiro, B. Skyline-plot methods for estimating demographic history from nucleotide sequences. *Mol. Ecol. Resour.* **2011**, *11*, 423–434. https://doi.org/10.1111/j.1755-0998.2011.02988.x.

2. Drummond, A. J. Bayesian coalescent inference of past population dynamics from molecular sequences. *Mol. Biol. Evol.* **2005**, *22*, 1185–1192. https://doi.org/10.1093/molbev/msi103.

3. Heled, J.; Drummond, A. J. Bayesian inference of population size history from multiple loci. *BMC Evol. Biol.* **2008**, *8*, 289. https://doi.org/10.1186/1471-2148-8-289.

4. Minin, V. N.; Bloomquist, E. W.; Suchard, M. A. Smooth skyride through a rough skyline: Bayesian coalescent-based inference of population dynamics. *Mol. Biol. Evol.* **2008**, *25*, 1459–1471. https://doi.org/10.1093/molbev/msn090.

5. Gill, M. S.; Lemey, P.; Faria, N. R.; Rambaut, A.; Shapiro, B.; Suchard, M. A. Improving Bayesian population dynamics inference: a coalescent-based model for multiple loci. *Mol. Biol. Evol.* **2013**, *30*, 713–724. https://doi.org/10.1093/molbev/mss265.

6. Suchard, M. A.; Lemey, P.; Baele, G.; Ayres, D. L.; Drummond, A. J.; Rambaut, A. Bayesian phylogenetic and phylodynamic data integration using BEAST 1.10. *Virus Evol.* **2018**, *4*, vey016. https://doi.org/10.1093/ve/vey016.

7. Ayres, D. L.; Cummings, M. P.; Baele, G.; Darling, A. E.; Lewis, P. O.; Swofford, D. L.; Huelsenbeck, J. P.; Lemey, P.; Rambaut, A.; Suchard, M. A. BEAGLE 3: improved performance, scaling, and usability for a high-performance computing library for statistical phylogenetics. *Syst. Biol.* **2019**, *68,* 1052–1061. https://doi.org/10.1093/sysbio/syz020.

8. Rambaut, A.; Drummond, A. J.; Xie, D.; Baele, G.; Suchard, M. A. Posterior summarization in Bayesian phylogenetics using Tracer 1.7. *Syst. Biol.* **2018**, *67*, 901–904. https://doi.org/10.1093/sysbio/syy032.

9. Villanea, F. A.; Kitchen, A.; Kemp, B. M. Applications of Bayesian skyline plots and approximate Bayesian computation for human demography. *Hum. Biol.* **2019**, *91*, 279-296. https://doi.org/10.13110/humanbiology.91.4.04.

10. Jeffreys, H. Some tests of significance, treated by the theory of probability. *Math. Proc. Camb. Phil. Soc.* **1935**, *31*, 203–222. https://doi.org/10.1017/S030500410001330X.

11. Kass, R. E.; Raftery, A. E. Bayes Factors. *J. Am. Stat. Assoc.* **1995**, *90*, 773–795. https://doi.org/10.1080/01621459.1995.10476572.

12. Lartillot, N.; Philippe, H. Computing Bayes Factors using thermodynamic integration. *Syst. Biol.* **2006**, *55*, 195–207. https://doi.org/10.1080/10635150500433722.

13. Xie, W.; Lewis, P. O.; Fan, Y.; Kuo, L.; Chen, M.-H. Improving marginal likelihood estimation for Bayesian phylogenetic model selection. *Syst. Biol.* **2011**, *60*, 150–160. https://doi.org/10.1093/sysbio/syq085.

14. Pybus, O. G.; Rambaut, A.; Harvey, P. H. An integrated framework for the inference of viral population history from reconstructed genealogies. *Genetics* **2000**, *155*, 1429–1437. https://doi.org/10.1093/genetics/155.3.1429.

15. Strimmer, K.; Pybus, O. G. Exploring the demographic history of DNA sequences using the generalized skyline plot. *Mol. Biol. Evol.* **2001**, *18*, 2298–2305. https://doi.org/10.1093/oxfordjournals.molbev.a003776.

16. Opgen-Rhein, R.; Fahrmeir, L.; Strimmer, K. Inference of demographic history from genealogical trees using reversible jump Markov Chain Monte Carlo. *BMC Evol. Biol.* **2005**, *5*, 6. https://doi.org/10.1186/1471-2148-5-6.

17. Paradis, E.; Schliep, K. Ape 5.0: an environment for modern phylogenetics and evolutionary analyses in R. *Bioinformatics* **2019**, *35*, 526–528. https://doi.org/10.1093/bioinformatics/bty633.

18. R Core Team. R: a language and environment for statistical computing; R Foundation for Statistical Computing: Vienna, Austria, **2023**. https://www.R-project.org/.

19. Felsenstein, J. Estimating effective population size from samples of sequences: inefficiency of pairwise and segregating sites as compared to phylogenetic estimates. *Genet. Res.* **1992**, *59*, 139–147. https://doi.org/10.1017/S0016672300030354.

20. Pybus, O. G.; Rambaut, A. GENIE: estimating demographic history from molecular phylogenies. *Bioinformatics* **2002**, *18*, 1404–1405. https://doi.org/10.1093/bioinformatics/18.10.1404.

21. Xiang, F.; Dearlove, B.; Frost, S. genieR: An R package for inference of demographic history of phylogenies. *J. Open Source Softw.* **2019**, *4*, 634. https://doi.org/10.21105/joss.00634.

22. Weiss, G.; Von Haeseler, A. Inference of population history using a likelihood approach. *Genetics* **1998**, *149*, 1539–1546. https://doi.org/10.1093/genetics/149.3.1539.

23. Schmidt, H. A.; Von Haeseler, A.; Buschbom, J. pIPHULA—parallel inference of population parameters using a likelihood approach. *Bioinformatics* **2007**, *23*, 2636–2637. https://doi.org/10.1093/bioinformatics/btm391.

24. Darriba, D.; Taboada, G. L.; Doallo, R.; Posada, D. jModelTest 2: more models, new heuristics and parallel computing. *Nat. Methods* **2012**, *9*, 772. https://doi.org/10.1038/nmeth.2109.

25. Schmidt, H. A.; Strimmer, K.; Vingron, M.; Von Haeseler, A. TREE-PUZZLE: maximum likelihood phylogenetic analysis using quartets and parallel computing. *Bioinformatics* **2002**, *18*, 502–504. https://doi.org/10.1093/bioinformatics/18.3.502.

26. Kuhner, M. K. LAMARC 2.0: maximum likelihood and Bayesian estimation of population parameters. *Bioinformatics* **2006**, *22*, 768–770. https://doi.org/10.1093/bioinformatics/btk051.

27. Kuhner, M. K.; Smith, L. P. Comparing likelihood and Bayesian coalescent estimation of population parameters. *Genetics* **2007**, *175*, 155–165. https://doi.org/10.1534/genetics.106.056457.

28. Felsenstein, J.; Churchill, G. A. A Hidden Markov Model approach to variation among sites in rate of evolution. *Mol. Biol. Evol.* **1996**, *13*, 93–104. https://doi.org/10.1093/oxfordjournals.molbev.a025575.

29. Griffiths, R. C.; Tavare, S. Simulating probability distributions in the coalescent. *Theor. Popul. Biol.* **1994**, *46*, 131–159. https://doi.org/10.1006/tpbi.1994.1023.

30. Griffiths, R. C.; Tavaré, S. Sampling theory for neutral alleles in a varying environment. *Philos. Trans. R. Soc. Lond. B Biol. Sci.* **1994**, *344*, 403–410. https://doi.org/10.1098/rstb.1994.0079.

31. Griffiths, R. C.; Tavaré, S. Ancestral inference in population genetics. *Stat. Sci.* **1994**, *9*, 307–319. https://doi.org/10.1214/ss/1177010378.

32. Griffiths, R. C.; Tavaré, S. Unrooted genealogical tree probabilities in the infinitely-many-sites model. *Math. Biosci.* **1995**, *127*, 77–98. https://doi.org/10.1016/0025-5564(94)00044-Z.

33. Kimura, M. The number of heterozygous nucleotide sites maintained in a finite population due to steady flux of mutations. *Genetics* **1969**, *61*, 893–903. https://doi.org/10.1093/genetics/61.4.893.

Results of the MCMC sampling-based skyline plot analyses

In the BSP analyses using a normally distributed prior for the substitution rate with mean 0.01 and standard deviation 0.0025 (in substitutions per site per Ma), truncated to be positive (corresponding to a 95% interquantile range of 0.005-0.015), the estimated mean tree root height was 281 ka (median: 248 ka; 95% HPD interval: 61-571 ka) for the piecewise-constant model (ESS > 1500 for all parameters), and 264 ka (median: 232 ka; 95% HPD interval: 60-536 ka) for the piecewise-linear model (ESS > 2200 for all parameters). The respective BSPs for the two models are shown in Fig. S5, in which it can be observed that the rise of the population size curve at the left end of the plots never exceeds the 95% HPD limits along the rest of the graph [1,2], where the curve is essentially flat. The results of analyses using either a uniformly or exponentially distributed prior for the substitution rate exhibited a similar pattern of no significant population size changes (not shown). In the EBSP analyses using the same normally distributed prior for the substitution rate as above, the estimated mean tree root height was 363 ka (median: 331 ka; 95% HPD interval: 85-717 ka) for the stepwise model (ESS > 2200 for all parameters), and 341 ka (median: 304 ka; 95% HPD interval: 58-688 ka) for the linear model (ESS > 2800 for all parameters). For the 'population size changes' parameter in each model, the estimated mean was 0.867 (median and mode were 1, and the 95% HPD interval was 0-2) and 1.024 (median and mode were 1, and the 95% HPD interval was 0-3), respectively. The respective EBSPs for the two models are presented in Fig. S6. Results of analyses using a uniformly distributed prior for the substitution rate also did not indicate significant population size changes in recent demographic history (not shown). The skyride (ESS > 6500 for all parameters) and skygrid (ESS > 650 for all parameters) analyses also yielded the

same result (Fig. S7); their respective mean tree root height estimates were 72 ka (median: 66 ka; 95% HPD interval: 24-133 ka) and 277 ka (median: 245 ka; 95% HPD interval: 67-564 ka). In the analyses with the constant population size (uniform population size prior; ESS > 4700 for all parameters) and exponential growth (uniform population size and growth rate priors; ESS > 1500 for all parameters) models, the estimated mean tree root heights were 419 ka (median: 349 ka; 95% HPD interval: 111-809 ka) and 508 ka (median: 349 ka; 95% HPD interval: 103-814 ka). The 95% HPD interval of the posterior distribution of the exponential growth rate parameter ([-1.803, 4.018]) included zero, i.e. did not reject a constant population size. The skygrid model was the one with the highest log marginal likelihood estimate (stepping-stone sampling MLE = -1096; path sampling MLE = -1095), but not significantly different from that of the piecewise-linear version of the BSP (MLE = -1097 by both stepping-stone sampling and path sampling). Still using Bayes factors, the skygrid model had positive support against the piecewise-constant version of the BSP (stepping-stone sampling MLE = -1099; path sampling MLE = -1098) and very strong support (2 ln Bayes factor > 10) against other models.

References

1. Ho, S. Y. W.; Shapiro, B. Skyline-plot methods for estimating demographic history from nucleotide sequences. *Mol. Ecol. Resour.* **2011**, *11*, 423–434. https://doi.org/10.1111/j.1755-0998.2011.02988.x.

2. Grant, W. S. Problems and cautions with sequence mismatch analysis and Bayesian skyline plots to infer historical demography. *J. Hered.* **2015**, *106*, 333–346. https://doi.org/10.1093/jhered/esv020.

Table S1. Information on the samples and previously published *Cyt b* sequences of striped hyenas and outgroups used in this study.

Sample code	Country	Location	Haplotype (648 bp alignment)	Haplotype (340 bp alignment)	GenBank accession number	Reference
Hy1	Algeria	Chlef	H1	H1	PP328974	This study
Hy2	Algeria	Batna	H1	H1	PP328974	This study
Hy3	Algeria	Tlemcen	H1	H1	PP328974	This study
Hy4	Algeria	Tlemcen	H1	H1	PP328974	This study
Hy5	Algeria	Médéa	H1	H1	PP328974	This study
Hy6	Algeria	Bouïra	H1	H1	PP328974	This study
Hy7	Algeria	Tlemcen	H1	H1	PP328974	This study
Hy9	Algeria	Biskra	H1	H1	PP328974	This study
Hy10	Algeria	Chlef	H1	H1	PP328974	This study
Hy11	Algeria	Bouïra	H1	H1	PP328974	This study
Hy13	Algeria	Constantine	H1	H1	PP328974	This study
Hy16	Algeria	Bouïra	H1	H1	PP328974	This study
Hy18	Algeria	Tlemcen	H1	H1	PP328974	This study
Hy21	Algeria	Médéa	H1	H1	PP328974	This study
Hy22	Algeria	Batna	H1	H1	PP328974	This study
Hy27	Algeria	Batna	H1	H1	PP328974	This study
Hy28	Algeria	Tlemcen	H1	H1	PP328974	This study
Hy29	Algeria	Batna	H1	H1	PP328974	This study
Hy30	Algeria	Tlemcen	H1		PP328974	This study
Hy31	Algeria	Tlemcen	H1		PP328974	This study

Sample code	Country	Location	Haplotype (648 bp alignment)	Haplotype (340 bp alignment)	GenBank accession number	Reference
Hy32	Algeria	Tlemcen	H1	H1	PP328974	This study
Hy33	Algeria	Tlemcen	H1	H1	PP328974	This study
Hy34	Algeria	Tlemcen	H1	H1	PP328974	This study
Hy35	Algeria	Tlemcen	H1	H1	PP328974	This study
Belezma_YRhigi	Algeria	Batna		H1	= PP328974	This study
	Algeria	Algiers		H1	DQ157581	Rohland et al. 2005
	Libya	Tripoli		H1	DQ157585	Rohland et al. 2005
	Cameroon			H1	DQ157579	Rohland et al. 2005
	Angola	Luanda		H7	DQ157582	Rohland et al. 2005
	Tanzania	NE Tanzania		H4	DQ157583	Rohland et al. 2005
	Tanzania		H11	H5	AY048787	Albert 2001
	Somalia	Somaliland		H5	DQ157584	Rohland et al. 2005
Hyena_Amr1	Jordan	Qatraneh		H3	= KP789314	This study
Hyena_Amr2	Jordan	Abu Nusair		H3	= KP789314	This study
	Syria			H4	DQ157577	Rohland et al. 2005
	Greece	Thebes		H5	DQ157578	Rohland et al. 2005
00-01C7-1926	Saudi Arabia	Taif	H2		PP328975	This study
00-01C7-F7AE	Saudi Arabia	Taif		H2	= PP328975	This study
Phiri	Saudi Arabia	Taif	H2		PP328975	This study
Arnold	Oman		H3	H1	= MT893967	This study
Buwayter	Oman		H3	H1	= MT893967	This study

**Sample code**	**Country**	**Location**	**Haplotype** (648 bp alignment)	**Haplotype** (340 bp alignment)	**GenBank accession number**	**Reference**
Dopey	Oman		H3	H1	= MT893967	This study
Luna	Oman		H3	H1	= MT893967	This study
Sirius	Oman		H3	H1	= MT893967	This study
	Oman			H3	KP789314	Unpublished
Hh1_Massoud	Iran	Kerman	H4	H6	= MT893978	This study
20_5_7_Massoud	Iran	Kerman	H4	H6	= MT893978	This study
	Iran	Razavi Khorasan	H3		MT893967	Dadashi-Jourdehi et al. 2021
	Iran	Qorveh	H3		MT893969	Dadashi-Jourdehi et al. 2021
	Iran	Khash	H3		MT893970	Dadashi-Jourdehi et al. 2021
	Iran	Bushehr	H3		MT893972	Dadashi-Jourdehi et al. 2021
	Iran	Nishapur	H3		MT893973	Dadashi-Jourdehi et al. 2021
	Iran	Damghan	H3		MT893974	Dadashi-Jourdehi et al. 2021
	Iran	Khuzestan	H3		MT893975	Dadashi-Jourdehi et al. 2021
	Iran	Kerman	H3		MT893984	Dadashi-Jourdehi et al. 2021
	Iran	Qom	H3		MT893989	Dadashi-Jourdehi et al. 2021
	Iran	Qazvin	H3		MT893991	Dadashi-Jourdehi et al. 2021
	Iran	Tehran	H3		MT893993	Dadashi-Jourdehi et al. 2021
	Iran	Qom	H3		MT893994	Dadashi-Jourdehi et al. 2021
	Iran	Hamedan	H3		MT893997	Dadashi-Jourdehi et al. 2021
	Iran	Razavi Khorasan	H4		MT893978	Dadashi-Jourdehi et al. 2021
	Iran	Razavi Khorasan	H4		MT893979	Dadashi-Jourdehi et al. 2021

Sample code	Country	Location	Haplotype (648 bp alignment)	Haplotype (340 bp alignment)	GenBank accession number	Reference
	Iran	Sabzevar	H4		MT893983	Dadashi-Jourdehi et al. 2021
	Iran	Joghatai	H4		MT893988	Dadashi-Jourdehi et al. 2021
	Iran	North Khorasan	H4		MT893990	Dadashi-Jourdehi et al. 2021
	Iran	Bardsir	H4		MT893996	Dadashi-Jourdehi et al. 2021
	Iran	Khatam	H4		MT893998	Dadashi-Jourdehi et al. 2021
	Iran	Bijar	H4		MT893999	Dadashi-Jourdehi et al. 2021
	Iran	Kerman	H4		MT894000	Dadashi-Jourdehi et al. 2021
	Iran	Khatam	H4		MT894001	Dadashi-Jourdehi et al. 2021
	Iran	Bam	H4		MT894002	Dadashi-Jourdehi et al. 2021
	Iran	Yasuj	H5		MT893965	Dadashi-Jourdehi et al. 2021
	Iran	Ardestan	H5		MT893966	Dadashi-Jourdehi et al. 2021
	Iran	Ardabil	H5		MT893968	Dadashi-Jourdehi et al. 2021
	Iran	East Azerbaijan	H5		MT893971	Dadashi-Jourdehi et al. 2021
	Iran	Khamir	H5		MT893976	Dadashi-Jourdehi et al. 2021
	Iran	Fars	H5		MT893977	Dadashi-Jourdehi et al. 2021
	Iran	Semnan	H5		MT893981	Dadashi-Jourdehi et al. 2021
	Iran	East Azerbaijan	H5		MT893985	Dadashi-Jourdehi et al. 2021
	Iran	Ardabil	H5		MT893987	Dadashi-Jourdehi et al. 2021
	Iran	Hamedan	H5		MT893992	Dadashi-Jourdehi et al. 2021
	Iran	Razavi Khorasan	H6		MT893980	Dadashi-Jourdehi et al. 2021
	Iran	Taft	H7		MT893982	Dadashi-Jourdehi et al. 2021

**Sample code**	**Country**	**Location**	**Haplotype** (648 bp alignment)	**Haplotype** (340 bp alignment)	**GenBank accession number**	**Reference**
	Iran	Khatam	H8		MT893986	Dadashi-Jourdehi et al. 2021
	Iran	Shahdad	H9		MT893995	Dadashi-Jourdehi et al. 2021
	Iran	Hamedan	H10		MT894003	Dadashi-Jourdehi et al. 2021
	Azerbaijan			H1	DQ157587	Rohland et al. 2005
	Russia	Vladikavkaz		H1	DQ157576	Rohland et al. 2005
	Russia	Siberia		H1	DQ157586	Rohland et al. 2005
	India			H1	DQ157580	Rohland et al. 2005
		Tierpark Berlin	H5	H3	AY048788	Albert 2001
		San Diego Zoo	H5	H3	AY928678	Koepfli et al. 2006
		Cerza Zoo	H5	H3	JF894376	Bon et al. 2012
**Outgroups**						
Parahyaena brunnea				Parahyaena_brunnea	NC038159	Westbury et al. 2018
Crocuta crocuta				Crocuta_crocuta	NC020670	Bon et al. 2012

a)

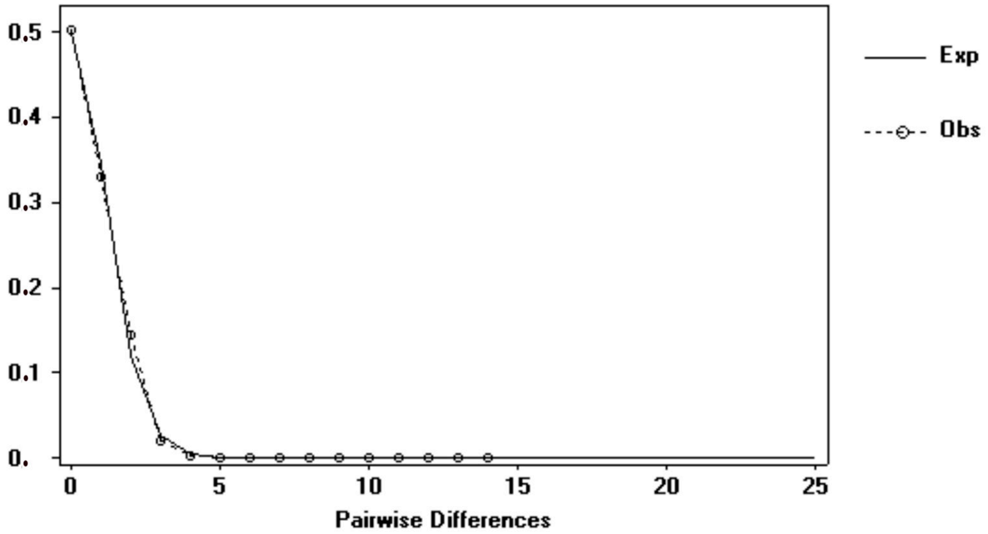

b)

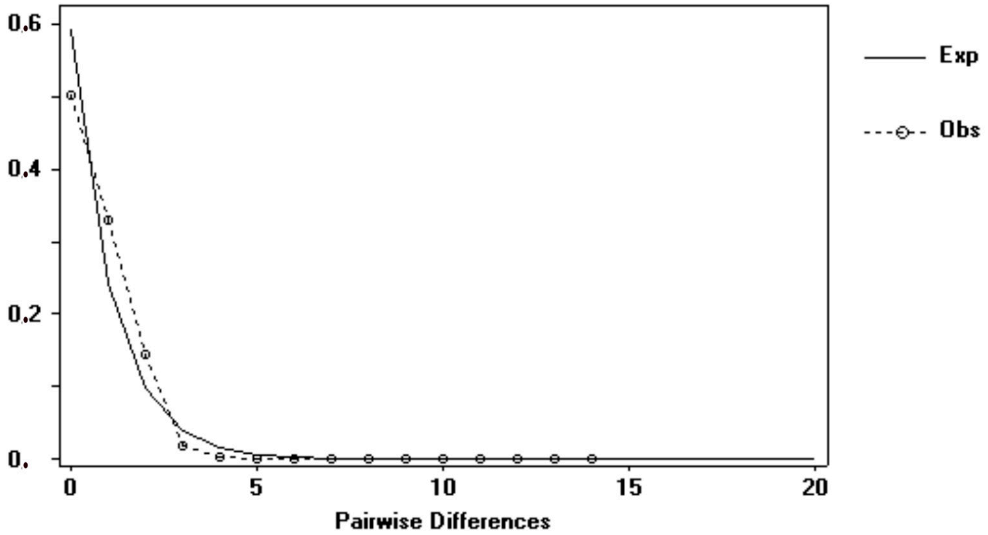

Figure. S1. DNASP graphs of mismatch distributions for the striped hyena 340 bp dataset comparing the observed distribution with (a) the expected values under a model of population growth or decline (Rogers and Harpending 1992), and (b) the expected values under a model of constant population size. The x-axis measures the number of pairwise nucleotide differences between sequences, while the y-axis measures the frequency of such pairwise differences in the dataset.

a)

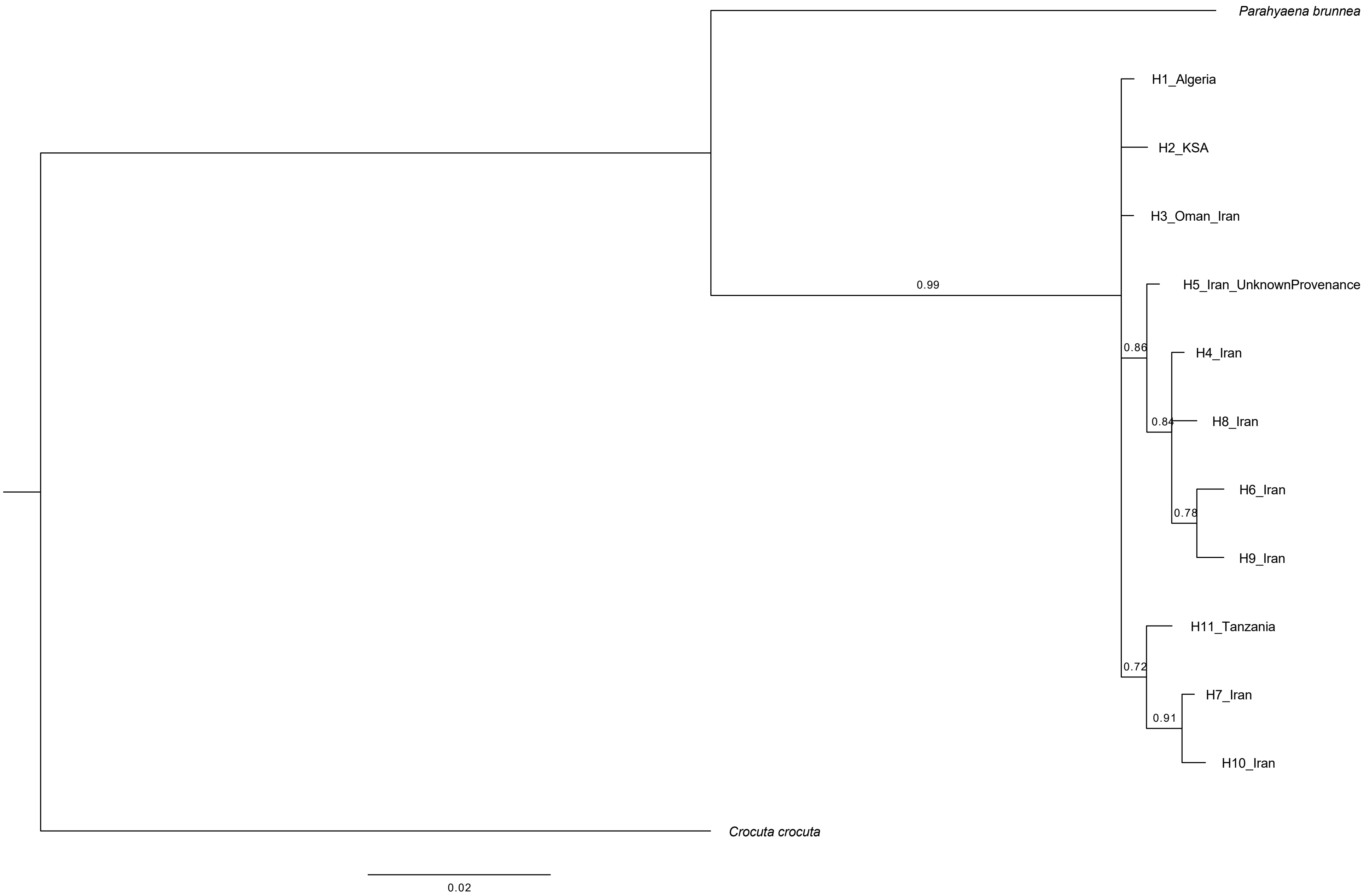
Parahyaena brunnea
H1_Algeria
H2_KSA
H3_Oman_Iran
0.99
H5_Iran_UnknownProvenance
0.86
H4_Iran
0.84
H8_Iran
H6_Iran
0.78
H9_Iran
H11_Tanzania
0.72
H7_Iran
0.91
H10_Iran
Crocuta crocuta
0.02

b)

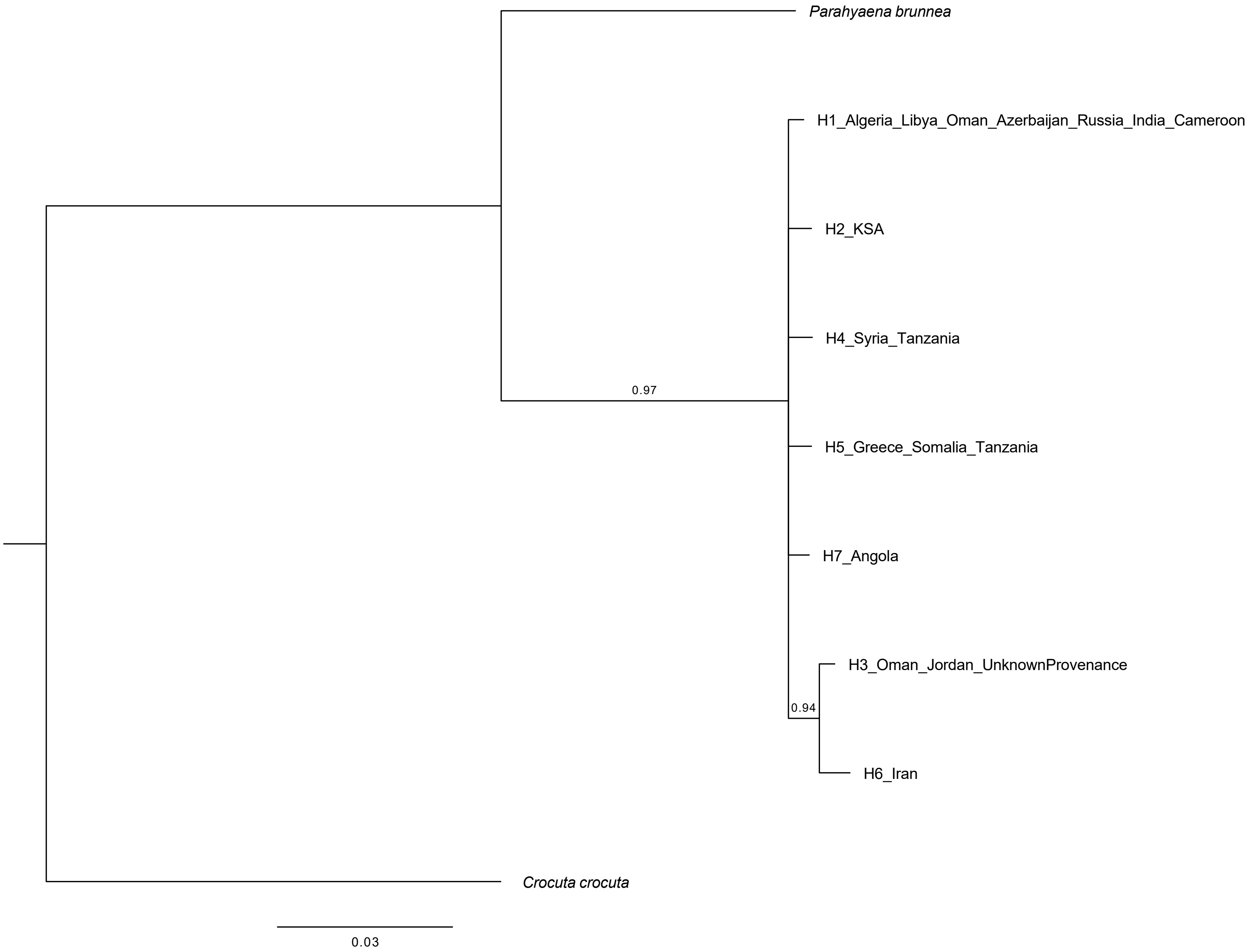
Parahyaena brunnea
H1_Algeria_Libya_Oman_Azerbaijan_Russia_India_Cameroon
H2_KSA
H4_Syria_Tanzania
0.97
H5_Greece_Somalia_Tanzania
H7_Angola
H3_Oman_Jordan_UnknownProvenance
0.94
H6_Iran
Crocuta crocuta
0.03

Figure. S2. Majority-rule consensus phylograms (cut-off 0.7) from the Bayesian inference analyses of the (a) 648-bp and (b) 340-bp *Cyt b* datasets, with two out-groups (*Parahyaena brunnea* and *Crocuta crocuta*). See Table S1 for haplotype information. Numbers above branches are Bayesian posterior probabilities.

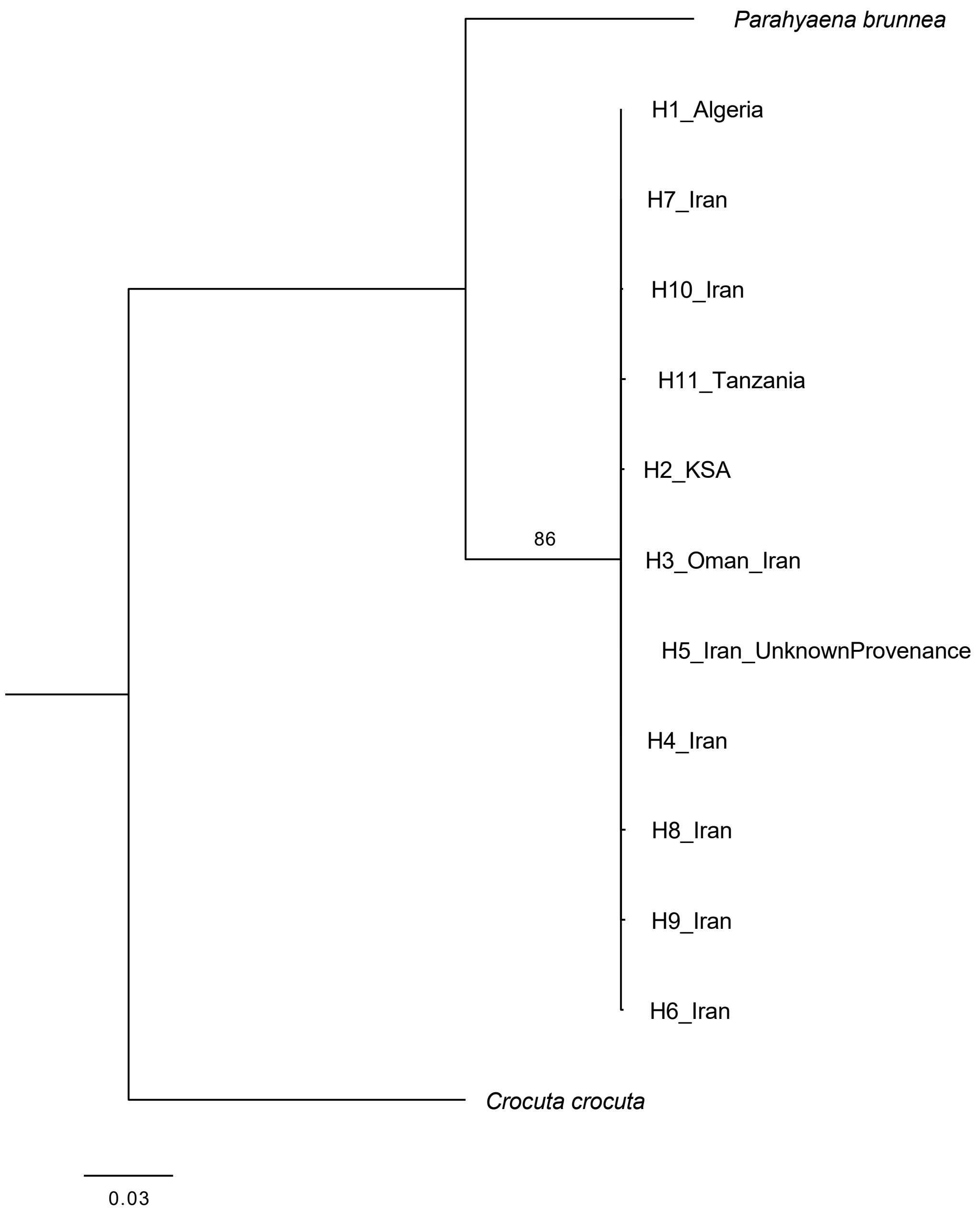

Figure. S3. Majority-rule consensus phylogram (cut-off 70%) of the maximum likelihood analysis of the 648 bp dataset with two outgroups (*Parahyaena brunnea* and *Crocuta crocuta*). See Table S1 for haplotype information. Numbers above branches are bootstrap values.

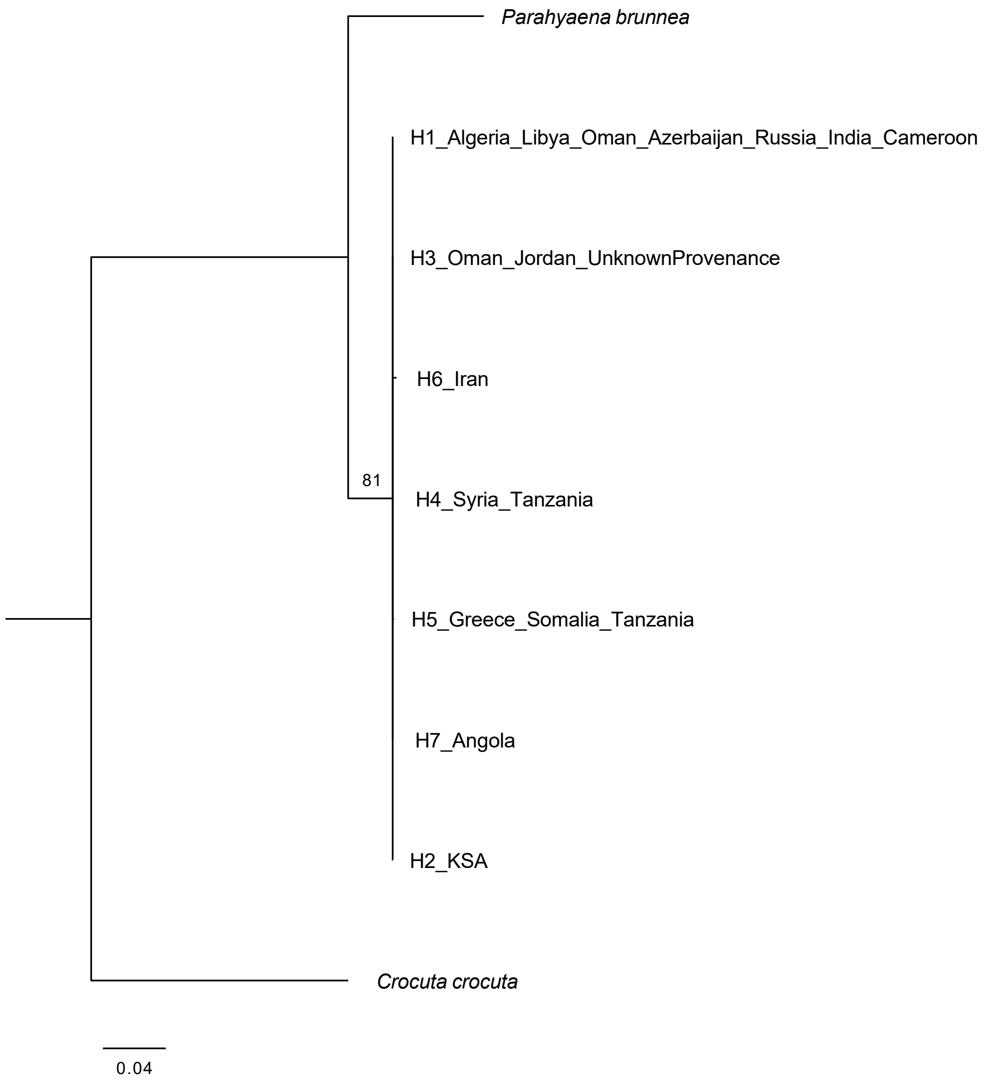

Figure. S4. Majority-rule consensus phylogram (cut-off 70%) of the maximum likelihood analysis of the 340 bp dataset with two outgroups (*Parahyaena brunnea* and *Crocuta crocuta*). See Table S1 for haplotype information. Numbers above branches are bootstrap values.

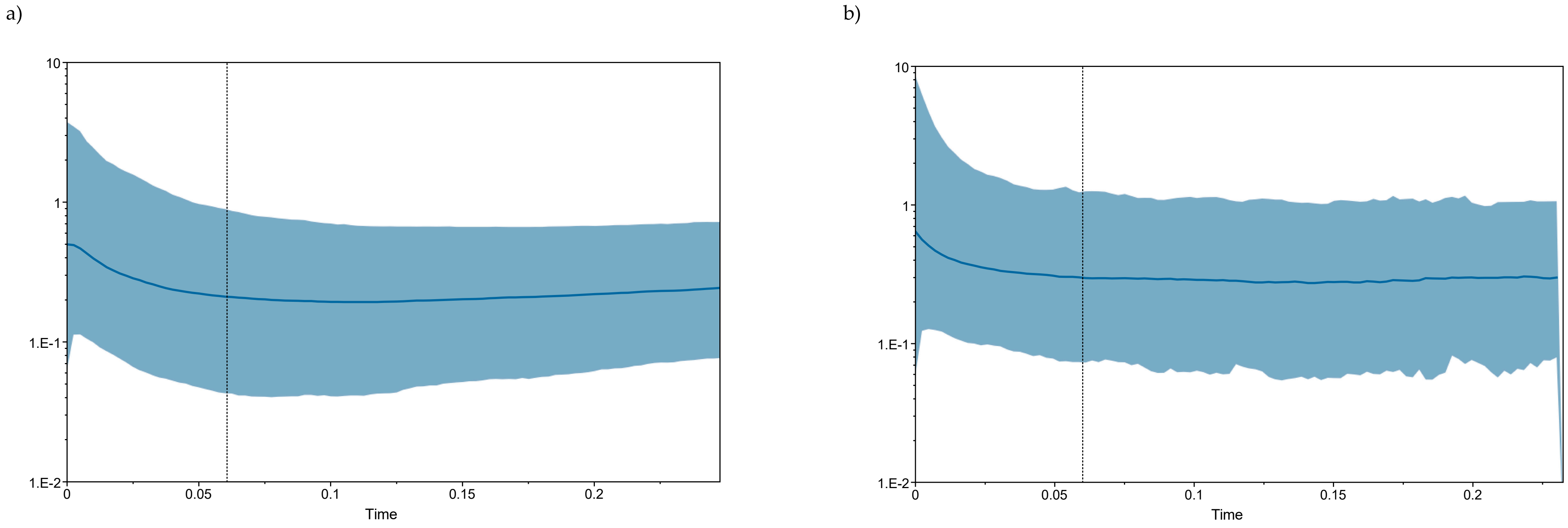

Figure. S5. BSPs for the sample of 76 striped hyena 648-bp *Cyt b* sequences, using the a) piecewise-constant and b) piecewise-linear models, and a normally distributed prior for the substitution rate with mean 0.01 and standard deviation 0.0025 (in substitutions per site per Ma). The thicker solid dark blue line is the median estimate of population size, and the lighter blue shaded region represents the 95% HPD envelope. Maximum time is the root height median, and the black vertical dotted line indicates the lower 95% HPD estimate. The x-axis (in linear scale) measures time in Ma units (going backwards in time from left to right) and the y-axis (in logarithmic scale) measures population size in units of estimated effective population size (N_e) x generation time in years x 10^{-6}.

a) b)

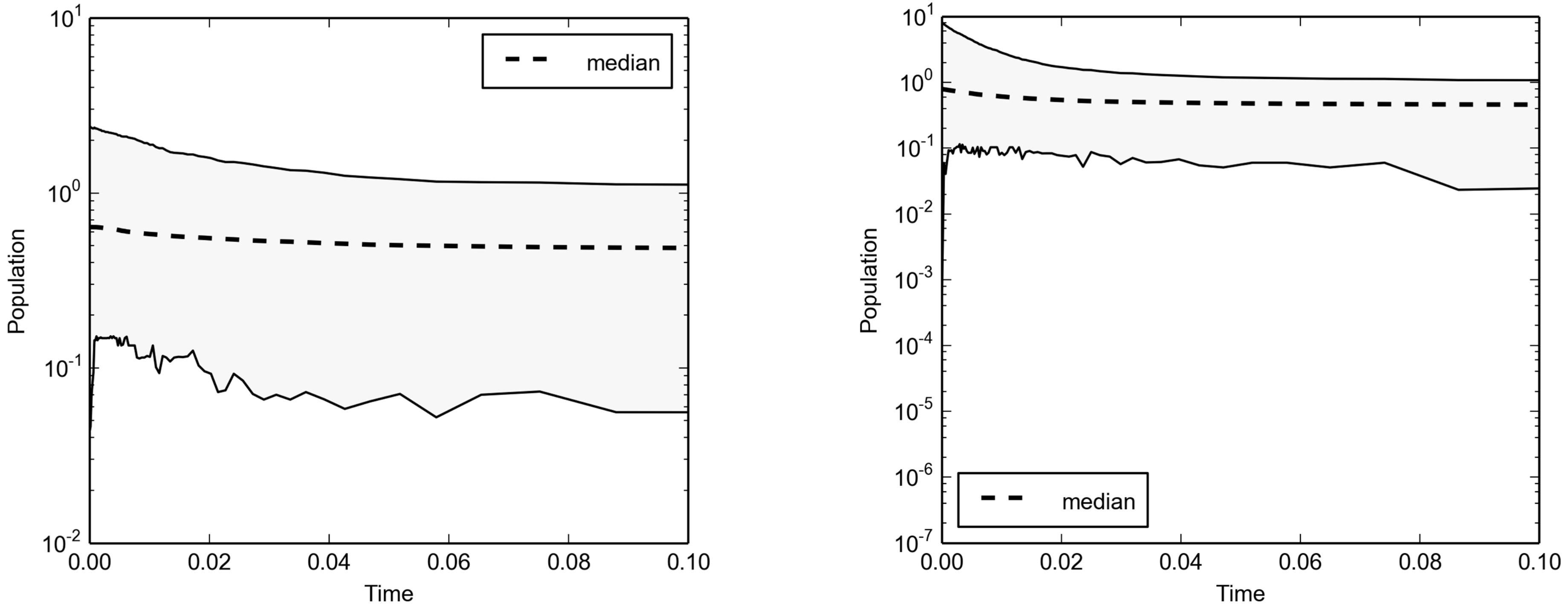

Figure. S6. EBSPs for the sample of 76 striped hyena 648-bp *Cyt b* sequences, using the a) stepwise and b) linear models, and a normally distributed prior for the substitution rate with mean 0.01 and standard deviation 0.0025 (in substitutions per site per Ma). The thicker dashed line is the median estimate of population size, and the shaded region represents the 95% HPD envelope. The x-axis (in linear scale) measures time in Ma units (going backwards in time from left to right) and the y-axis (in logarithmic scale) measures population size in units of estimated effective population size (N_e) x generation time in years x 10^{-6}.

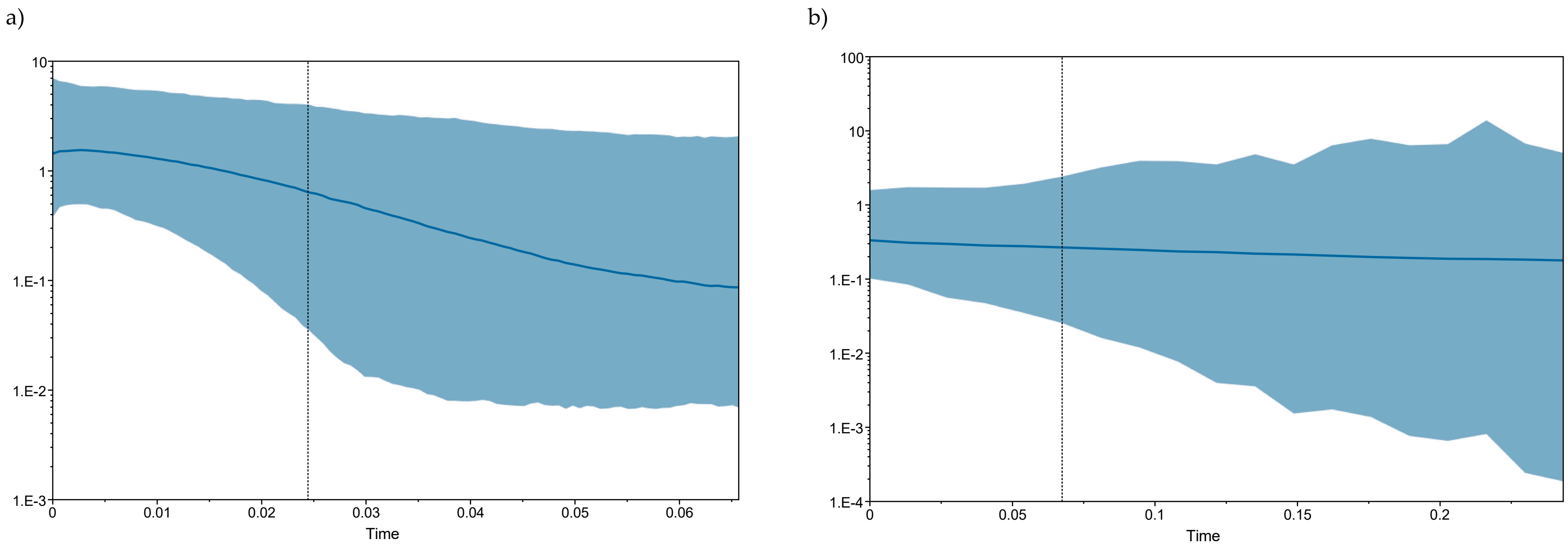

Figure. S7. Skyline plots for the sample of 76 striped hyena 648-bp *Cyt b* sequences, using the a) skyride and b) skygrid methods, and a normally distributed prior for the substitution rate with mean 0.01 and standard deviation 0.0025 (in substitutions per site per Ma). The thicker solid dark blue lines are the median estimates of population size, and the lighter blue shaded regions represent 95% HPD envelopes. Maximum time is the root height median, and the black vertical dotted lines indicate the lower 95% HPD estimates. The x-axis (in linear scale) measures time in Ma units (going backwards in time from left to right) and the y-axis (in logarithmic scale) measures population size in units of estimated effective population size (N_e) x generation time in years x 10^{-6}.

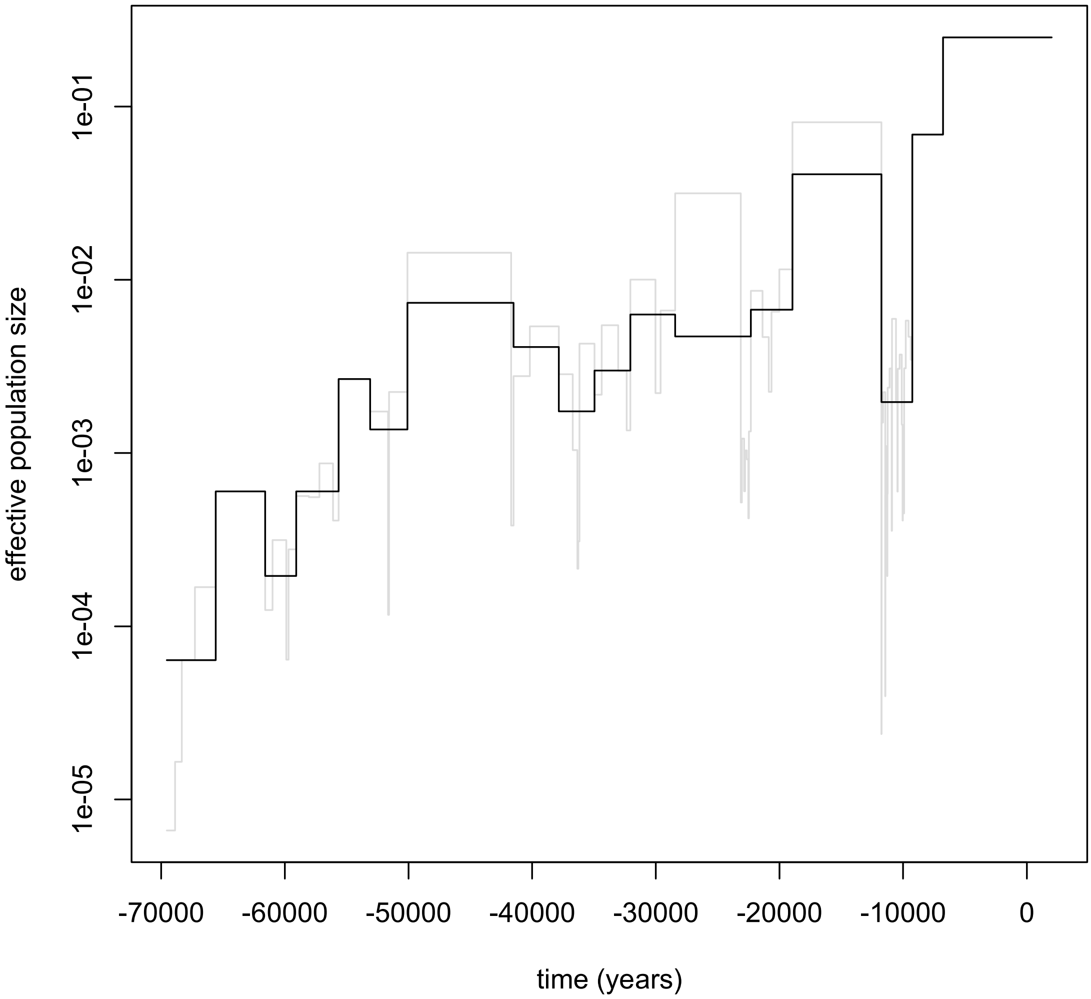

Figure. S8. Generalized skyline plot (black line), and classic skyline plot (grey line) in the background. Time is zero at the present.

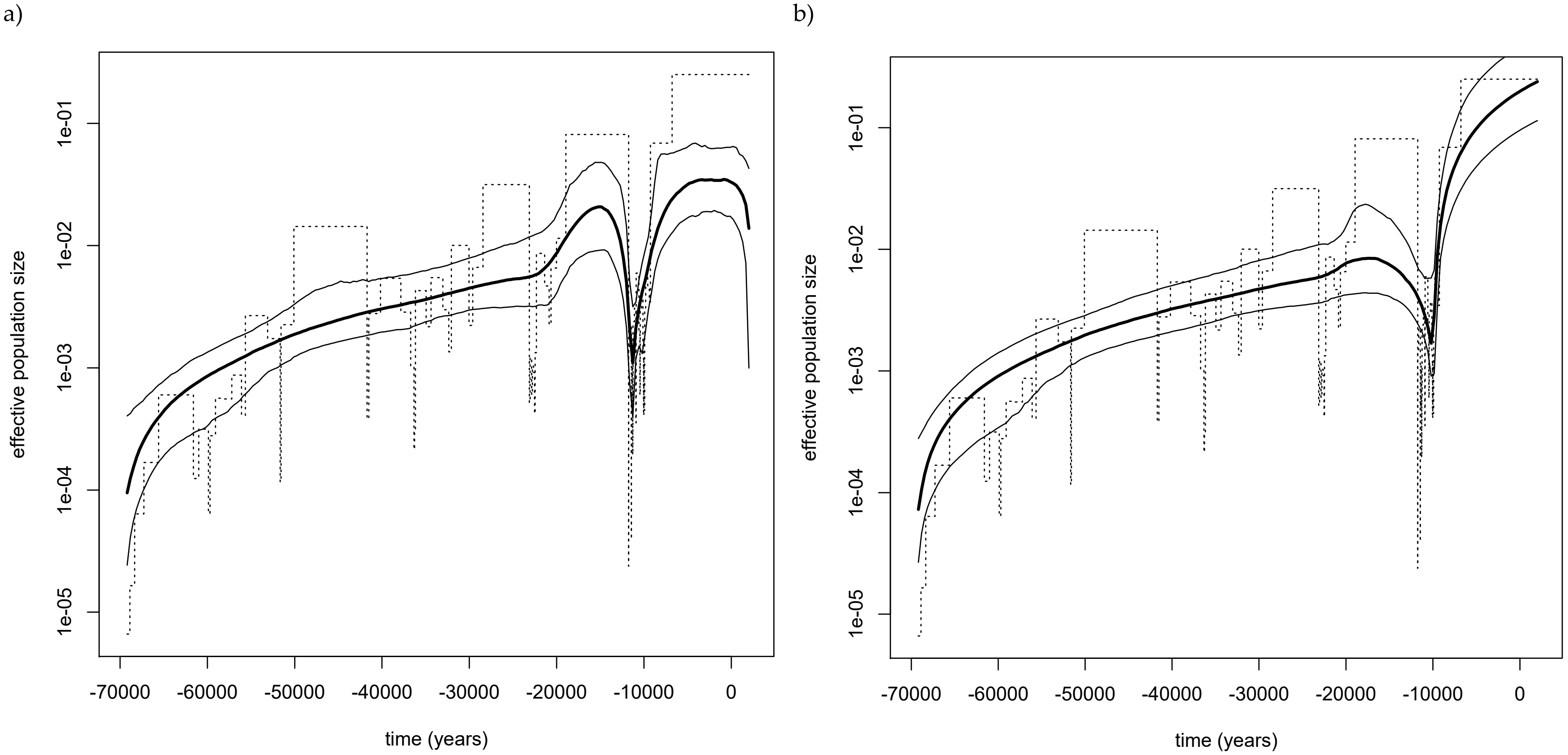

Figure. S9. Bayesian MCP plots estimated using a) a constant population size prior and b) a skyline plot prior. The thicker black lines are the median of the posterior distribution of population size, and the thinner lines represent the 95% confidence intervals. In both figures, for comparison, the classic skyline plot is shown in the background (dashed lines). Time is zero at the present.
